# Childhood trauma and relationship with the parents: associations with lipids, glucose, and prolactin levels during antipsychotic treatment in patients with first-episode psychosis

**DOI:** 10.3389/fpsyt.2025.1627203

**Published:** 2025-08-14

**Authors:** Marianne Laffely, Claire Grosu, Nermine Laaboub, Philippe Golay, Luis Alameda, Philippe Conus, Chin B. Eap

**Affiliations:** ^1^ Unit of Pharmacogenetics and Clinical Psychopharmacology, Department of Psychiatry, Centre for Psychiatric Neuroscience, Lausanne University Hospital, University of Lausanne, Prilly, Switzerland; ^2^ Department of Psychiatry, Service of General Psychiatry, Lausanne University Hospital, University of Lausanne, Prilly, Switzerland; ^3^ La Source School of Nursing, Haute Ecole spécialisée de Suisse occidentale (HES-SO) University of Applied Sciences and Arts, Lausanne, Switzerland; ^4^ Service of General Psychiatry, Treatment and Early Intervention in Psychosis Program, Lausanne, University Hospital (CHUV), Lausanne, Switzerland; ^5^ Department of Psychosis Studies, Institute of Psychiatry, Psychology and Neuroscience, King’s College of London, London, United Kingdom; ^6^ Centro Investigacion Biomedica en Red de Salud Mental (CIBERSAM); Instituto de Biomedicina de Sevilla (IBIS), Hospital Universitario Virgen del Rocio, Departamento de Psiquiatria, Universidad de Sevilla, Sevilla, Spain; ^7^ University of Lausanne, Lausanne, Switzerland; ^8^ Les Toises Psychiatry and Psychotherapy Center, Lausanne, Switzerland

**Keywords:** childhood trauma, first-episode psychosis, lipids, prolactin, antipsychotics, parental relationship

## Abstract

**Background:**

Besides antipsychotics and other clinical factors, childhood trauma (CT) may also alter metabolic and endocrine profiles. The aim was to determine whether CT influence metabolic profiles and prolactin levels in patients with first-episode psychosis treated with antipsychotics for up to one year. The quality of the relationship with the parents was also investigated.

**Methods:**

Two hundred twenty-six patients with low-density lipoprotein (LDL), high-density lipoprotein (HDL), non-HDL, total cholesterol, triglycerides, fasting plasma glucose, and prolactin levels monitored routinely before and during antipsychotic treatment were included. CT refers to physical, sexual, or emotional abuse occurring before the prodromal phase and up to 16 years old and was assessed during the patients’ 3 years follow-up. The quality of the relationship with parents was assessed at the beginning of the follow-up.

**Results:**

After 12 months, CT was associated with an increase in total and non-HDL cholesterol levels (+17%, 95%CI:[0.4; 34]; +11%, 95%CI:[0.4; 21], respectively). Lack of a good relationship with the father was associated at 2 and 12 months with above-median levels of LDL (OR=5.5, 95%CI:[1.3, 28]; OR=3.7, 95%CI:[1.3; 12], respectively) and non-HDL (OR=4.7, 95%CI:[1.3; 19]; OR=2.8, 95%CI:[1.0; 8.0], respectively). Lack of a good relationship with the mother was associated with higher baseline prolactin levels in women (124 ng/ml, 95%CI:[91; 157]) and increased total cholesterol levels after 2 and 12 months (21%, 95%CI:[5.9; 36]; 12%, 95%CI:[1.1; 24]), respectively).

**Conclusion:**

Childhood trauma or a lack of a good relationship with one parent is associated with worse metabolic and prolactin profiles in first-episode psychosis patients during the first year of antipsychotic treatment.

## 1 Introduction

Patients suffering from psychosis have more than 10-year shorter life expectancy compared to the general population (1). Cardiovascular diseases are the leading cause of premature death in this population and are partly due to antipsychotic medications (2). Metabolic alterations are also observed in drug-naïve patients (3) which may be explained by factors such as poor health habits, smoking, alcohol consumption, and a sedentary lifestyle (4). Although studies investigating the lipidic and glycaemic profiles in patients with first-episode psychosis (FEP) yielded heterogeneous results (5–9), some Genome-Wide Association Studies (GWAS) have found common genes between schizophrenia and cardiovascular risk factors (10, 11), magnifying the hypothesis of psychosis as a multisystem disorder. Of note, along with the growing interest in the role of epigenetics in the etiopathogenesis of mental health outcomes, childhood trauma (CT) has also been proposed as a risk factor for psychosis and for the development of metabolic alterations (12–14). The association between CT and poor outcomes in psychosis has been widely studied (15–17). Thus, in a large meta-analysis, CT has been found to increase the odds of psychosis by 2.8 (18) and the prevalence of CT among the population with psychosis varies from 30% to 60% (19). CT is also associated with more extreme positive and depressive symptoms as well as poorer functional outcomes in people with psychosis (15–17). Adverse childhood experiences have also been linked to metabolic alterations. Recent results from the 30-year longitudinal CARDIA study show a higher prevalence of type 2 diabetes mellitus and hyperlipidaemia in subjects reporting a CT (12). CT may therefore partially explain the high rates of metabolic abnormalities in the FEP population. However, only a few studies have analysed the role of childhood trauma on clinical and metabolic markers in FEP and, while outcomes tend to point out metabolic alterations, there are discrepancies related to specific metabolic markers involved (20–23).

Of note, in FEP, metabolic parameters are not the only biological system affected; endocrine imbalances have also been described and may even precede metabolic alterations. Thus, although hyperprolactinaemia is a side effect of many antipsychotics (24), a growing number of studies demonstrated that hyperprolactinaemia is also present in drug-naïve FEP patients (25–27) even before the first episode, i.e., in patients who are at risk for psychosis (28, 29), suggesting that elevated prolactin could also be a risk factor for psychosis.

Similarly to metabolic alterations, prolactin imbalance could therefore be associated with CT (25). Stresses such as CT can stimulate the release of prolactin, which is partially regulated through the secretion of prolactin-inhibiting factor, a product similar to dopamine (30). Although the biological pathways linking stress and psychosis are complex and multifactorial, alterations in prolactin regulation may represent one of the mechanisms contributing to this association (25). Moreover, prolactin acts directly on the hypothalamic–pituitary–adrenal (HPA) axis, inducing higher levels of cortisol (31). This suggests a possible indirect effect of prolactinaemia on the advent of psychosis and metabolic alterations since cortisol is a trigger for both psychosis and metabolic abnormalities (32). Hence, higher levels of prolactin and cortisol induced by a history of CT could contribute to the higher rates of metabolic alterations in FEP patients. However, although cortisol has been widely investigated in the association between CT and FEP, little is known about prolactin in FEP patients with CT.

Besides trauma, insecure attachment is also associated with psychosis (33, 34). The attachment theory suggests that adulthood interpersonal relationships are related to the nature of the bond with the caregiver in early childhood, and remain mostly stable across the lifespan (33). However, metabolic consequences of the quality of parental care in childhood have been poorly studied, and results are scarce (35, 36). Similarly, little is known about the effect of attachment style and endocrine alterations. Prolactinaemia has been found to be elevated in subjects reporting poor quality of relationships with their parents (37, 38).

Taken together, trauma and the quality of parental care may partially contribute to the high rates of metabolic abnormalities in the FEP population. However, although there is growing interest in the association between trauma, psychosis, and metabolic alterations (5, 23, 39), there is a lack of studies examining metabolic and endocrine profiles in the FEP population with a history of CT.

The present study aimed to examine the association of CT in the form of sexual, physical, and emotional abuse and the quality of the relationship with parents at the beginning of follow-up with biological profiles in terms of lipidaemia, fasting plasma glucose (FPG), and prolactinaemia in an early psychosis population at the beginning and during the first year of antipsychotic treatment. Given the above, we believe that CT and poor relationships with parents have a negative impact on the above-mentioned metabolic and endocrine variables before and during antipsychotic treatment.

## 2 Materials and methods

### 2.1 Procedure and subjects

TIPP (Treatment and Early Intervention in Psychosis Program), a specialized early psychosis program, was launched in 2004 at the Department of Psychiatry, Lausanne University Hospital Switzerland (40). Entry criteria to the program are: [I] aged between 18 and 35; [II] residing in the catchment area [Lausanne and surroundings; population about 300 000]; [III] meeting threshold criteria for psychosis, as defined by the “Psychosis threshold” subscale of the Comprehensive Assessment of At Risk Mental States [CAARMS Scale (40, 41). In the TIPP program, each patient is followed up by a psychiatrist and a Case manager. The program involves multiple meetings over 3 years between patients and their case managers, as well as scheduled regular appointments with psychiatrists. The Ethics Committee of the Canton of Vaud (CER-VD) granted access to TIPP clinical data (demographic, assessment of trauma history, levels of symptoms, and other clinical variables). More details on the TIPP program can be found elsewhere (21).

In the Department of Psychiatry at Lausanne University Hospital, patients aged 18 years old or older starting a psychotropic treatment that is known to have a potential risk of inducing metabolic disturbances (clozapine, olanzapine, risperidone, quetiapine, aripiprazole, amisulpride, lithium, valproate and/or mirtazapine) are prospectively followed up for metabolic parameter evolution (see the Biological measures section below). Baseline clinical data were obtained during hospitalization and follow‐up data were obtained in the hospital or in out‐patient centers during a medical examination based on the department guideline for metabolic follow‐up performed on a routine basis (42, 43). Since 2007, a study (PsyMetab) has been ongoing to determine the clinical, environmental, and genetic determinants of metabolic adverse effects of psychotropic drugs (44).

This observational prospective study has been approved by the ethics committee of the Canton de Vaud (CER-VD). Informed written consent was obtained for all participants. Data collected between 01.01.2007 and 06.08.2021 were extracted for the present study. In addition, the CER-VD granted access to the data of patients with a metabolic follow-up in the Department of Psychiatry, Lausanne University Hospital, collected before 01.01.2016 (PsyClin). The present analysis involves participants included in the TIPP program and who were also part of the PsyMetab or PsyClin study between 08.05.2007 until 31.12.2015 (PsyClin) and until 06.08.2021 (PsyMetab). A detailed description of PsyMetab and PsyClin can be found elsewhere (45).

### 2.2 Assessment of history of past trauma and quality of relationship with the father and the mother in the TIPP cohort

The assessments are described in detail elsewhere (21). In summary, CT is defined if at least one of the three abuses (sexual, physical, or emotional) has been experienced by the patient before the age of 16. History of childhood trauma is assessed by the case manager during the 3-year follow-up of the TIPP program. Details about assessment can be found in **Supplementary Informations**. Patients are asked at baseline about the quality of their relationship with each parent. This categorical variable ranges from 0 to 5 (0: absent parent; 1 to 5: very bad, bad, suitable, good, very good relationship, respectively). The number of subjects in each categorical variable was insufficient to run statistical analyses separately; therefore, this variable was dichotomized as good relationship with the father or mother (GRF or GRM, respectively; scores 3 to 5) and lack of good relationship with the father or mother (LGRF or LGRM respectively; scores 0 to 2).

### 2.3 Biological measures

PsyMetab and PsyClin collect metabolic (i.e., body mass index (BMI), fasting plasma glucose (FPG), triglycerides, total cholesterol (TC), and low-density lipoprotein (LDL) and high-density lipoprotein (HDL) cholesterol) and clinical (i.e., diagnosis, age, sex, and smoking status) data from patients’ medical records at the beginning of weight-gain-inducing psychotropic treatments, and at 2, 3 and 12 months. Additional metabolic observations can be obtained during the hospital stay. Because we aimed to focus on blood parameters, clinical parameters were not analysed but, rather, used as covariates in statistical models.

All results from all available blood samples were taken into account except for FPG and triglycerides, which were kept only in patients who were fasting (46). Non-HDL cholesterol was calculated (TC minus HDL cholesterol). The analysis was defined using the subsequent timepoints in relation to the initiation of a psychotropic treatment: 0 (-30 to +7 days), 2 (45 to 75 days), 3 (75 to 105 days), and 12 (290 to 430 days) months in order to reach a sufficient number of patients at each timepoint to run statistical analysis. For prolactin determinations, samples from PsyMetab with no antipsychotic use at least 15 days before the introduction of an antipsychotic were used. Details about determinations of prolactin concentrations are described elsewhere (47). Hyperprolactinaemia was defined for men and women as ≥16.5 ng/ml and ≥28.3 ng/ml, respectively (48). Prolactinaemia, triglycerides, and FPG were analysed at baseline. Other time points were not analysed due to insufficient data availability. Baseline was defined up to 7 and up to 3 days after the onset of antipsychotic treatment for metabolic variables and for prolactin, respectively. In the case of an interruption of the psychotropic medication for more than two weeks or in the case of replacement by another drug, the follow-up is restarted from baseline. In the case of an introduction of a second drug from the list (see list in **Supplementary Table 1**), the follow-up is restarted. In such case, only the earliest follow-up, which has less missing information, was considered.

### 2.4 Other clinical variables

Gender refers to biological sex as recorded in official civil status documents. Participants were categorized as male or female based on this information. Ethnicity was dichotomized into “Westerners” and “Non Westerners” according to clinical data form Psymetab/PsyClin. When possible, missing values were filled in with data from the Genome-Wide Association Studies (see **Supplementary Information** for more details). Psychotropic medications were categorized into low, medium, and high risk of weight gain described in **Supplementary Table 1**. In order to adjust for the sum of the doses of antipsychotic intake per day, a variable was created named “weighting of the total daily dose of antipsychotic”. This variable weights the daily dose of an antipsychotic to its upper limit of the average dose per day adapted from Swissmedic (**Supplementary Table 2**). A sum of weightings of each antipsychotic taken in the same day is calculated. Medications were also categorized by their effect on prolactin levels based on a large meta-analysis (49) (see **Supplementary Table 3**). Socio-economic status was calculated according to an area-based index of Swiss socio-economic position (SSEP), ranging between 0 (most disadvantaged) and 100 (most privileged). The characterization of the SSEP index by place of residency was based on 2000 census data including income, education, occupation, and housing conditions. To estimate patients’ SES, postal addresses were obtained and geocoded using Google API via the ggmap R package. More details can be found elsewhere (50).

### 2.5 Statistical analysis

Descriptive data are shown as numbers and percentages for categorical variables or as median and interquartile range for continuous variables. For comparisons between groups, t-tests, Wilcoxon-Mann-Whitney rank-sum tests, or the Chi-squared test were used, depending on the variable type. For multivariate analyses, generalized linear models and linear mixed-effects models were used to examine the associations between plasma levels of prolactin and metabolic parameters with CT or the relationship with the mother or the father. Backward selection was performed on each model to keep only the significant covariates, resulting in different covariate adjustments between models (see **Supplementary List 1** for full models).

Data preparation was conducted using Stata 17.0 (StataCorp; College Station, Texas). Univariate and multivariate analyses were performed in R environment (version 4.1.2; Rstudio, Inc; Boston, Massachusetts). Statistical significance was determined at α = 0.05.

## 3 Results

### 3.1 Demographic and biological results

A total of 226 subjects were included in both PsyMetab and TIPP cohorts. The demographic and clinical parameters are shown in **Table 1**, while metabolic parameters are displayed in **Table 2**. Of note, no significant associations between biological variables and the outcomes were found at 3 months and were therefore not included in the tables and figures. Considering the percentage of missing values for biological parameters in **Table 2**, the number of dropouts after 1 year was greater in the groups reporting no CT, GRM, and GRF. The number of dropouts was also reported after 2 months in the group reporting GRM.

**Table 1:** Clinical parameters at baseline and demographic variables.

	Childhood trauma (CT)			Relationship with the father			Relationship with the mother		
	Yes	No	P-value	Lack of good relationship (LGRF)	Good relationship (GRF)	P-value	Lack of good relationship (LGRM)	Good relationship (GRM)	P-value
	(N=61; 27%)	(N=165; 73%)		(N=70; 32.6%)	(N=145; 57.4%)		(N=40; 18.5%)	(N=176; 81.5%)	
Age of onset of psychosis (years) Median (Q1-Q3)	25 (20.0 - 29.0)	22 (19.0 - 27.0)	**0.02**	23 (19.3 - 28.0)	22 (19.0 - 27.0)	0.19	23 (19.0 - 27.3)	23 (19.0 - 27.0)	0.81
Age at entry in follow-up (years) Median (Q1-Q3)	27 (22.0 - 30.0)	23 (20.0 - 28.0)	**0.01**	25 (22.0 - 29.0)	23 (20.0 - 28.0)	0.1	25 (21.8 - 29.0)	24 (20.0 - 28.0)	0.35
Men, N(%)	33 (54.1%)	112 (67.9%)	0.08	44 (62.9%)	95 (65.5%)	0.82	30 (75.0%)	109 (61.9%)	0.17
Socio-economic status, N(%)*									
Low	13 (21.3%)	34 (20.6%)	0.84	20 (28.6%)	24 (16.6%)	0.11	8 (20.0%)	37 (21.0%)	0.92
Medium	28 (45.9%)	70 (42.4%)		29 (41.4%)	65 (44.8%)		17 (42.5%)	79 (44.9%)	
High	20 (32.8%)	61 (37.0%)		21 (30.0%)	56 (38.6%)		15 (37.5%)	60 (34.1%)	
Westerners, Yes N(%)	39 (63.9%)	121 (73.3%)	0.2	49 (70.0%)	104 (71.7%)	0.86	27 (67.5%)	127 (72.2%)	0.65
Missing	0 (0%)	1 (0.6%)		0 (0%)	1 (0.7%)		0 (0%)	1 (0.6%)	
Post-school education, N(%)									
Apprenticeship	21 (34.4%)	40 (24.2%)	**0.02**	19 (27.1%)	40 (27.6%)	0.77	8 (20.0%)	53 (30.1%)	0.54
Professional school	6 (9.8%)	5 (3.0%)		4 (5.7%)	7 (4.8%)		1 (2.5%)	10 (5.7%)	
University	4 (6.6%)	28 (17.0%)		7 (10.0%)	20 (13.8%)		6 (15.0%)	23 (13.1%)	
Missing	30 (49.2%)	92 (55.8%)		40 (57.1%)	78 (53.8%)		25 (62.5%)	90 (51.1%)	
Marital status, N(%)									
Single	41 (67.2%)	143 (86.7%)	0.08	53 (75.7%)	125 (86.2%)	0.09	34 (85.0%)	144 (81.8%)	0.43
Married	7 (11.5%)	13 (7.9%)		5 (7.1%)	12 (8.3%)		2 (5.0%)	15 (8.5%)	
Divorced	3 (4.9%)	2 (1.2%)		3 (4.3%)	1 (0.7%)		2 (5.0%)	3 (1.7%)	
Cohabiting	5 (8.2%)	7 (4.2%)		6 (8.6%)	5 (3.4%)		1 (2.5%)	10 (5.7%)	
Missing	5 (8.2%)	0 (0%)		3 (4.3%)	2 (1.4%)		1 (2.5%)	4 (2.3%)	
BMI at baseline (kg/m^2^) Median (Q1-Q3)	22 (20.0 - 25.9)	22 (19.6 - 25.1)	0.38	23 (20.2 - 26.0)	22 (19.7 - 25.2)	0.28	23 (20.6 - 26.0)	22 (19.8 - 25.2)	0.81
Missing	2 (3.3%)	10 (6.1%)		3 (4.3%)	8 (5.5%)		1 (2.5%)	11 (6.3%)	
Waist circumference (cm) Median (Q1-Q3)	84 (74.5 - 89.8)	81 (75.0 - 92.0)	0.74	84 (76.8 - 93.0)	82 (75.0 - 90.0)	0.49	84 (76.8 - 91.8)	84 (74.0 - 90.5)	0.4
Missing	27 (44.3%)	71 (43.0%)		34 (48.6%)	57 (39.3%)		16 (40.0%)	77 (43.8%)	
Smokers, Yes N(%)	39 (63.9%)	120 (72.7%)	0.26	45 (64.3%)	106 (73.1%)	0.24	28 (70.0%)	125 (71.0%)	1
Alcohol, Yes N(%)	34 (55.7%)	97 (58.8%)	0.54	40 (57.1%)	87 (60.0%)	0.76	25 (62.5%)	101 (57.4%)	0.72
Missing	0 (0%)	7 (4.2%)		2 (2.9%)	5 (3.4%)		1 (2.5%)	6 (3.4%)	
Metabolic risk for psychotropic drugs**, N(%)									
Low risk	11 (18.0%)	19 (11.5%)	0.48	11 (15.7%)	19 (13.1%)	0.73	6 (15.0%)	24 (13.6%)	0.96
Medium risk	18 (29.5%)	44 (26.7%)		17 (24.3%)	40 (27.6%)		13 (32.5%)	45 (25.6%)	
High risk	7 (11.5%)	24 (14.5%)		11 (15.7%)	19 (13.1%)		6 (15.0%)	23 (13.1%)	
Missing	25 (41.0%)	78 (47.3%)		31 (44.3%)	67 (46.2%)		15 (37.5%)	84 (47.7%)	

Significant p-value in bold.

*Classification of socio-economic status in Switzerland was performed regarding Swiss socio-economic position (SSEP). Patients’ addresses were obtained and then classified according to SSEP (50).

**Psychotropic medications were categorized into low, medium and high-risk of metabolic alterations (see **Supplementary Table 1**).

N, number; BMI, body mass index.

**Table 2:** Metabolic parameters over time.

	Childhood Trauma (CT)			Relationship with the father			Relationship with the mother		
	Yes	No	P-value	Lack of good relationship (LGRF)	Good relationship (GRF)	P-value	Lack of good relationship (LGRM)	Good relationship (GRM)	P-value
	(N=61; 27%)	(N=165; 73%)		(N=70; 32.6%)	(N=145; 57.4%)		(N=40; 18.5%)	(N=176; 81.5%)	
LDL cholesterol baseline (mmol/l)									
Median (Q1-Q3)	2.4 (1.8 - 3.2)	2.4 (1.8 - 2.9)	0.25	2.5 (1.9 - 3.2)	2.3 (1.7 - 2.9)	**0.045**	2.2 (1.7 - 3.3)	2.5 (1.8 - 3.0)	0.85
Missing	17 (27.9%)	76 (46.1%)		28 (40.0%)	58 (40.0%)		13 (32.5%)	74 (42%)	
LDL cholesterol 2 months (mmol/l)									
Median (Q1-Q3)	2.7 (1.9 - 3.1)	2.6 (2.0 - 3.1)	0.62	3.0 (2.2 - 3.3)	2.4 (1.9 - 2.8)	**0.047**	2.7 (2.4 – 3.4)	2.4 (1.8 - 3.0)	0.12
Missing	47 (77.1%)	117 (70.9%)	0.86	48 (68.6%)	110 (75.9%)	0.17	23 (57.5%)	134 (76.1%)	**0.02**
LDL cholesterol 12 months (mmol/l)									
Median (Q1-Q3)	**3.2** (2.4 - 3.4)	2.7 (2.2 - 3.2)	0.1	**3.1** (2.6 - 3.4)	2.7 (2.2 - 3.3)	0.21	**3.1** (2.5 - 3.3)	2.8 (2.2 - 3.3)	0.62
Missing	31 (50.8%)	111 (67.3%)	**0.02**	38 (52.8%)	99 (68.3%)	**0.03**	18 (45%)	120 (68.18%)	**<0.01**
HDL cholesterol baseline (mmol/l)									
Median (Q1-Q3)	1.4 (1.0 - 1.7)	1.3 (1.1 - 1.5)	0.41	1.3 (1.0 - 1.5)	1.3 (1.1 - 1.6)	0.54	1.3 (1.1 - 1.4)	1.3 (1.1 - 1.6)	0.15
Missing	16 (26.2%)	75 (45.5%)		27 (38.6%)	57 (39.3%)		12 (30.0%)	73 (41.5%)	
HDL cholesterol 2 months (mmol/l)									
Median (Q1-Q3)	1.5 (1.2 - 1.6)	1.3 (1.1 - 1.5)	0.44	1.3 (1.1 - 1.5)	1.3 (1.1 - 1.5)	0.79	1.3 (1.1 - 1.4)	1.3 (1.1 - 1.6)	0.81
Missing	47 (77.1%)	116 (70.3%)	0.88	48 (68.6%)	109 (75.2%)	0.19	23 (57.5%)	133 (75.6%)	**0.02**
HDL cholesterol 12 months (mmol/l)									
Median (Q1-Q3)	1.3 (1.0 - 1.5)	1.3 (1.0 - 1.5)	0.63	1.4 (1.0 - 1.6)	1.3 (1.1 - 1.5)	0.13	1.3 (1.1 - 1.5)	1.3 (1.0 - 1.5)	0.85
Missing	31 (50.8%)	108 (65.5%)	**0.02**	37 (52.9%)	97 (66.9%)	**0.03**	18 (45%)	117 (66.48%)	**0.01**
Total cholesterol baseline (mmol/l)									
Median (Q1-Q3)	4.2 (3.6 - 5.2)	4.3 (3.6 - 5.0)	0.35	4.5 (3.8 - 5.3)	4.1 (3.4 - 4.8)	**0.03**	4.1 (3.5 - 5.3)	4.4 (3.6 - 5.0)	0.93
Missing	16 (26.2%)	68 (41.2%)		24 (34.3%)	53 (36.6%)		12 (30%)	66 (37.5%)	
Total cholesterol 2 months (mmol/l)									
Median (Q1-Q3)	4.4 (4.1 - 4.8)	4.4 (3.8 - 5.2)	0.8	4.8 (4.0 - 5.3)	4.3 (3.7 - 4.8)	0.18	4.4 (4.1 - 5.4)	4.4 (3.7 - 4.9)	0.25
Missing	46 (75.4%)	115 (69.7%)	0.84	48 (68.6%)	107 (73.8%)	0.26	23 (57.5%)	131 (74.43%)	**0.03**
Total cholesterol 12 months (mmol/l)									
Median (Q1-Q3)	**5.2** (4.2 - 5.6)	4.4 (4.0 - 5.3)	**0.04**	**5.2** (4.4 - 5.4)	4.4 (4.0 - 5.4)	0.06	**5.0** (4.3 - 5.4)	4.7 (4.1 - 5.4)	0.6
Missing	31 (50.8%)	107 (64.9%)	**0.04**	37 (52.9%)	96 (66.2%)	**0.04**	18 (45.0%)	116 (65.9%)	**0.01**
Non-HDL cholesterol baseline (mmol/l)									
Median (Q1-Q3)	2.8 (2.1 - 3.9)	2.8 (2.2 - 3.4)	0.38	3.1 (2.5 - 4.2)	2.8 (2.1 - 3.4)	**0.04**	2.8 (2.1 – 4.0)	2.9 (2.2 - 3.6)	0.59
Missing	16 (26.2%)	75 (45.5%)		27 (38.6%)	57 (39.3%)		12 (30%)	73 (41.5%)	
Non-HDL cholesterol 2 months (mmol/l)									
Median (Q1-Q3)	3.2 (2.4 - 3.4)	3.0 (2.6 - 3.7)	0.98	3.4 (2.6 - 3.9)	2.9 (2.4 - 3.2)	0.14	3.2 (2.9 - 4.0)	3.1 (2.4 - 3.6)	0.23
Missing	47 (77.1%)	116 (70.3%)	0.88	48 (68.6%)	109 (75.2%)	0.19	23 (57.5%)	133 (75.57%)	**0.02**
Non-HDL cholesterol 12 months (mmol/l)									
Median (Q1-Q3)	3.7 (3.2 - 4.2)	3.1 (2.7 - 3.8)	0.07	3.7 (3.1 - 4.2)	3.4 (2.8 - 3.9)	0.39	3.6 (2.9 - 3.9)	3.4 (2.8 - 4.3)	0.83
Missing	31 (50.8%)	108 (65.5%)	**0.03**	37 (52.9%)	97 (66.9%)	**0.03**	18 (45.0%)	117 (66.5%)	**0.01**
Prolactin baseline (ng/ml)									
Median (Q1-Q3)	20 (14.3 - 64.0)	23 (17.1 - 32.4)	0.83	20.5 (14.6 - 55.0)	24.0 (16.1 - 38.5)	0.66	24.8 (14.9 - 53.0)	22.2 (16.4 - 41.0)	0.77
Missing	40 (65.6%)	119 (72.1%)		47 (67.1%)	102 (70.3%)		31 (77.5%)	119 (67.6%)	
FPG baseline (mmol/l)									
Median (Q1-Q3)	5.0 (4.7 - 5.4)	4.8 (4.5 - 5.1)	0.17	4.8 (4.4 - 5.6)	4.8 (4.6 - 5.2)	0.52	4.8 (4.6 - 5.0)	4.8 (4.5 - 5.2)	0.99
Missing	34 (55.7%)	103 (62.4%)		47 (67.1%)	84 (57.9%)		23 (57.5%)	108 (61.4%)	
Triglycerides baseline (mmol/l)									
Median (Q1-Q3)	0.94 (0.79 - 1.2)	0.88 (0.64 - 1.3)	0.49	0.97 (0.63 - 1.3)	0.9 (0.7 - 1.2)	0.58	1.1 (0.63 - 1.2)	0.9 (0.7 - 1.2)	0.98
Missing	38 (62.3%)	112 (67.9%)		52 (74.3%)	90 (62.1%)		26 (65.0%)	118 (67.0%)	

Significant p-value in bold.

Physiological range for metabolic parameters: LDL cholesterol <3 mmol/L; HDL cholesterol >1 mmol/L; total cholesterol <5 mmol/L; Non-HDL cholesterol <3.8 mmol/L; triglycerides < 1.7 mmol/L (46). Median values above or below physiological ranges are indicated in bold/underlined.

LDL, low-density lipoprotein; HDL, high-density lipoprotein; Non-HDL, non-high-density lipoprotein; FPG, Fasting plasma glucose.

Median levels of TC were above the physiological range at 12 months in the CT, LGRF and LGRM groups (5.2 mmol/L, 5.2 mmol/L, and 5.0 mmol/L,respectively; **Table 2**), but not in patients with no CT and in those with GRF and GRM (4.4 mmol/L, 4.4 mmol/L and 4.7 mmol/L, respectively). Median levels of LDL were above the physiological range at 12 months in the CT, LGRF and LGRM groups (3.2 mmol/L, 3.1 mmol/L and 3.1 mmol/L, respectively) but not in patients with no CT, with GRF and with GRM (2.7 mmol/L, 2.7 mmol/L, 2.8 mmol/L, respectively).

### 3.2 Childhood trauma

**Figure 1:**
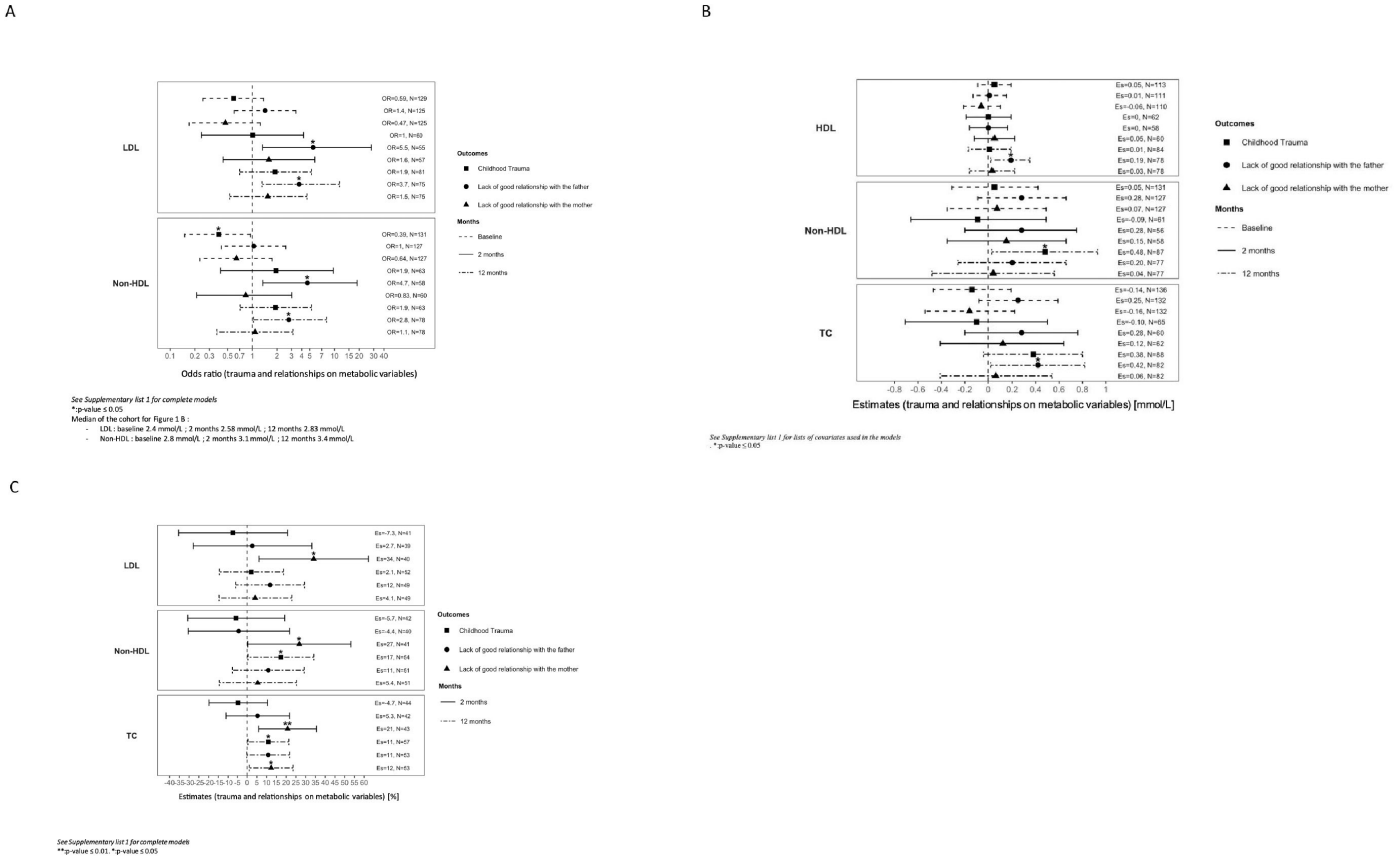
**(A)** Effects of trauma and relationship on metabolic variables over time. Variables were analysed on a categorical scale dichotomized by the median of the cohort at baseline and at 2 and 12 months of follow-up. Median of the cohort for (A): - LDL: baseline 2.4 mmol/L; 2 months 2.58 mmol/L; 12 months 2.83 mmol/L. - Non-HDL: baseline 2.8 mmol/L; 2 months 3.1 mmol/L; 12 months 3.4 mmol/L See Supplementary List 1 for lists of covariates used in models. OR, odds ratio; LDL, low-density lipoprotein; Non- HDL, non-high density lipoprotein; N, number of subjects. *** p-value < 0.001; ** p-value ≤= 0.01. *p-value ≤ 0.05. **(B)** Associations of trauma and relationship with metabolic variables over time. Variables were analysed at baseline, and at 2 and 12 months of follow-up. See Supplementary List 1 for lists of covariates used in models. Es, estimates; HDL, high-density lipoprotein; Non-HDL, non-high density lipoprotein; TC, total cholesterol; N, number of subjects. * p-value ≤ 0.05. **(C)** Percentage of change from baseline of metabolic variables regarding trauma and relationship over time. Footnotes: Variables were analysed once on a continuous scale. Percentage of difference in metabolic parameters between groups (CT and non-CT, quality of relationship with the parent) was analysed between baseline and at 2, 3, and 12 months of follow-up. See **Supplementary List 1** for lists of covariates used in models. Es, estimates; LDL, low-density lipoprotein; Non-HDL, non-high density lipoprotein; TC, total cholesterol; N, number of subjects. ** p-value ≤ 0.01. * p-value ≤ 0.05.

CT in the forms of sexual, physical, and emotional abuse occurred in 27% of the cohort. Patients with CT were older at the beginning of symptoms (25 y vs 22 y, p=0.02; **Table 1**) and at entry into the cohort (27 y vs 23 y, p=0.01). CT was associated with a decreased risk of having non-HDL cholesterol level above physiological values (2.8 mmol/L) at baseline (OR=0.39, 95%CI:[0.2; 0.95], **Figure 1A**). CT was associated with higher levels of non-HDL at 12 months (0.48 mmol/L, 95%CI:[0.03; 0.93]; **Figure 1B**) with a 17% higher increase (95%CI:[0.4;34]; **Figure 1C**) compared to the non-CT group after multivariate analysis. TC was significantly higher in patients with CT (5.2 mmol/L vs 4.4 mmol/L, p<0.05; **Table 2**), which increased by 11% (95%CI:[0.4;21]; **Figure 1C**) at 12 months in the CT group as compared to the non-CT group. No associations were found between CT and LDL, HDL, FPG, or triglycerides levels.

### 3.3 Relationship with the father

LGRF occurred in 33% of the included patients (**Table 1**). LDL levels were significantly higher in patients reporting LGRF (2.5 mmol/L vs 2.3 mmol/L, p<0.05; **Table 2**). At 2 months, LGRF patients presented elevated LDL (3.0 mmol/L vs 2.4 mmol/L, p<0.05), and in multivariate analysis, LGRF was significantly associated with approximately a 5-fold increased risk of having LDL levels above the median after 2 months (median=2.58 mmol/L, OR=5.5, 95%CI:[1.3;28]; **Figure 1A**) and approximately a 4-fold increased risk after one year of follow-up (median=2.8 mmol/L, OR=3.7, 95%CI:[1.3;12]). After one year of treatment, LGRF was significantly associated with a higher level of HDL (0.19, 95%CI:[0.02;0.35] mmol/L; **Figure 1B**). Non-HDL at baseline was significantly higher in patients reporting LGRF (3.1 mmol/L vs 2.8 mmol/L, p=0.04; **Table 2**). Similar to results with LDL, LGRF was significantly associated in multivariate analysis with approximately a 5-fold increased risk of having non-HDL levels above the median after 2 months (median=3.1 mmol/L, OR=4.7, 95%CI:[1.3; 19]; **Figure 1A**) and approximately a 3-fold increased risk after one year (median=3.4 mmol/L, OR=2.8, 95%CI:[1.03; 8.0]).

TC at baseline was significantly higher in patients reporting LGRF (4.5 mmol/L vs 4.1 mmol/L, p=0.03; **Table 2**). After one year of treatment, LGRF was significantly associated with a higher level of TC (0.42, 95%CI:[0.02; 0.82] mmol/L; **Figure 1B**).

Finally, no association was found between LGRF with prolactin, FPG, or triglycerides.

### 3.4 Relationship with the mother

In univariate analysis, no significant differences in terms of demographic or biological results were found between patients with and without GRM (**Tables 1**, **2**). In multivariate analysis, LGRM was associated with an increase of LDL by 34% after 2 months (95%CI[6.1; 62]; **Figure 1C**) and with an increase of TC by 21% (95%CI=[5.9; 36]) and 12% (95%CI:[1.1; 24]) after 2 months and one year, respectively. No association was found for HDL, FPG, or triglycerides.

### 3.5 Analysis of prolactin levels stratified by gender

**Figure 2:**
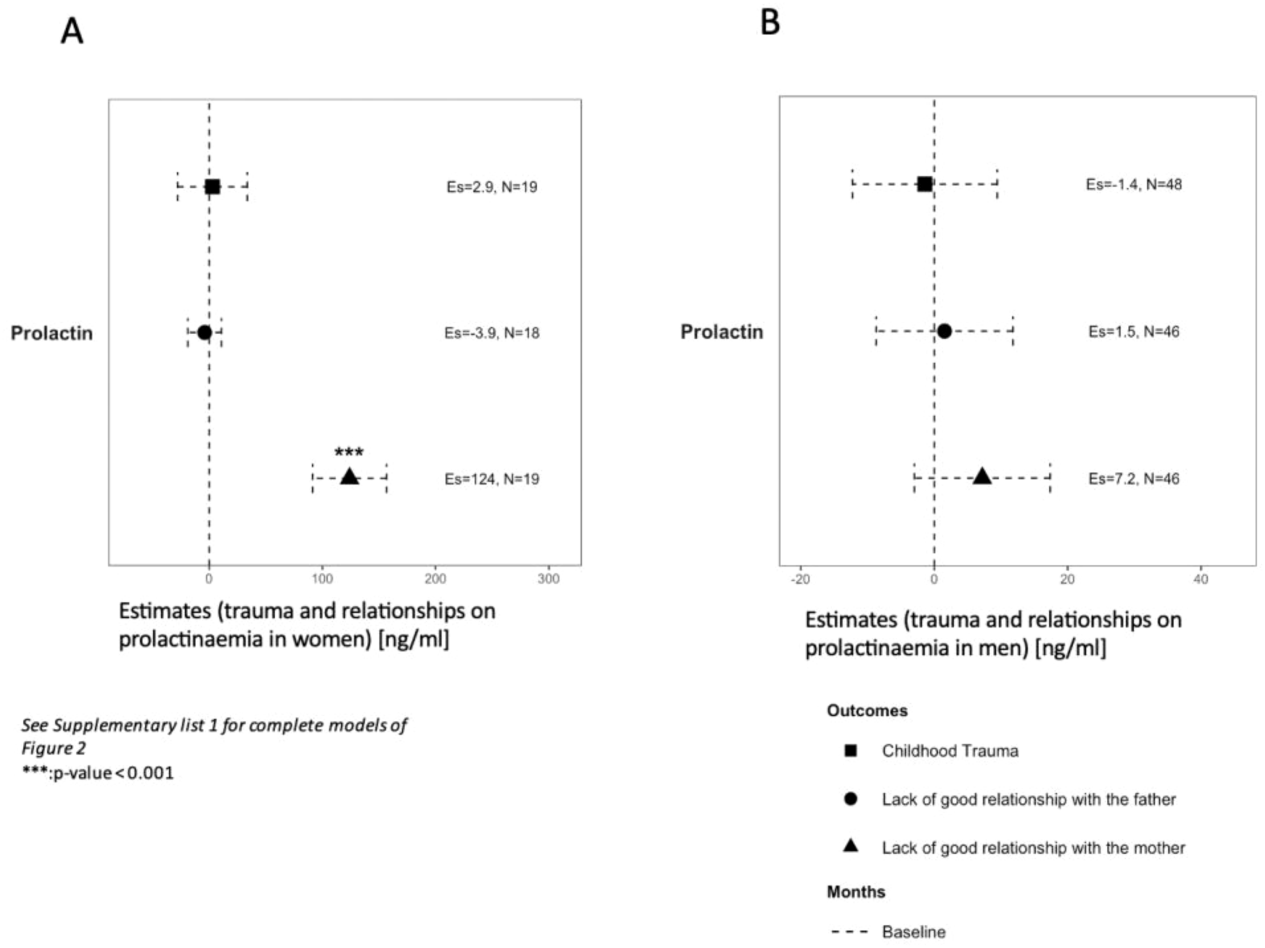
Association of trauma and relationships with prolactinaemia at baseline in women **(A)** and men **(B)**. Variables were analysed on a continuous scale at baseline in two groups regarding a different physiological median in each sex. See Supplementary List 1 for lists of covariates used in models. Es, estimates. *** p-value < 0.001.

Levels of prolactin at baseline were above the physiological threshold in men regardless of history of CT and quality of relationship with both parents (**Table 3**). In women, levels of prolactin were also above threshold except for LGRF and LGRM (28 ng/ml and 21 ng/ml, respectively). In multivariate analysis, levels of prolactin at baseline were elevated in women reporting LGRM (124, 95%CI:[91, 157]ng/ml, p<0.001; **Figure 2A**) but not in men (7.2, 95%CI:[-3; 17] ng/mL; **Figure 2B**).

**Table 3:** Prolactin values at baseline stratified by gender.

		Childhood Trauma (CT)			Relationship with the father			Relationship with the mother		
		Yes	No	P-value	Lack of good relationship (LGRF)	Good relationship (GRF)	P-value	Lack of good relationship (LGRM)	Good relationship (GRM)	P-value
		(N=61; 28 W, 33 M)	(N=165; 53 W, 112 M)		(N=70; 26 W, 44 M)	(N=145; 50 W, 95 M)		(N=40; 10 W, 30 M)	(N=176; 67 W, 109 M)	
Women	Prolactin baseline (ng/ml)									
	Median (Q1-Q3)	**51** (25.5 - 79.3)	**41** (22.2 - 49.0)	0.83	28 (24.6 - 49.0)	**41** (23.2 - 72.0)	0.66	22 (14.0 - 24.4)	42 (25.7 - 75.8)	0.77
	Missing	22 (78.6%)	40 (75.5%)		21 (80.8%)	36 (72%)		7 (70%)	51 (76.1%)	
Men	Prolactin baseline (ng/ml)									
	Median (Q1-Q3)	**19** (17.9 - 24.3)	**20** (12.1 - 30.7)	0.83	**20** (15.4 - 26.0)	**19** (13.1 - 30.7)	0.66	**26** (17.1 - 45.4)	**19** (14.0 - 30.0)	0.77
	Missing	20 (60.6%)	77 (68.8%)		27 (61.4%)	64 (67.4%)		23 (76.7%)	68 (62.4%)	

W, women; M, men; Physiological range for prolactin values: men ≤16.5 ng/ml; women ≤28.3 ng/ml. Median values above physiological ranges are indicated in bold/underlined.

Finally, multivariate analyses for lipidaemia, prolactin, and FPG were conducted combining CT with LGRF or LGRM to examine whether or not lack of good relationships with parents would worsen those values in patients with CT, but no significant results were found (data not shown).

## 4 Discussion

Overall, CT, LGRM, and LGRF were associated with worse metabolic profile after the introduction of an antipsychotic but not before. To the best of our knowledge, this is the first study looking at associations between CT and metabolic effects throughout the first year after the first episode of psychosis. These results are in line with a previous study of first-episode schizophrenia showing higher LDL and total cholesterol levels in patients with CT (22) and are also consistent with those of a 3-year-prospective longitudinal study assessing metabolic parameters in a FEP cohort receiving antipsychotics, during which lipid parameters were mostly increased during the first year of follow-up (51). The present study suggests that CT, LGRF and LGRM increase this phenomenon.

To our knowledge, the present study is also the first to investigate the association between CT and prolactinaemia in a FEP population. No association was found between CT and prolactin levels at baseline. However, as expected, median baseline levels of prolactin were above physiological threshold in both genders and are consistent with previous studies measuring prolactinaemia in FEP populations (26, 48). LGRM was associated with higher levels of prolactinemia at baseline in women in our study. Hyperprolactinaemia is frequently found in FEP patients and could be an epiphenomenon of an acute stress response, stress triggering prolactin release (30, 32, 52). However, hyperprolactinaemia in a group at risk of mental illness and later developing psychosis raises the question of the aetiopathogenic role of prolactin in the onset of psychosis (29). The current model of psychosis development involves an increase in dopamine mediators through intracerebral mesolimbic and mesocortical pathways. However, animal studies suggest that prolactin has a direct effect on the mesolimbic dopamine system, which is involved in the pathophysiology of psychosis (53, 54). Furthermore, the tuberoinfundibular pathway also secretes dopamine to inhibit prolactin secretion; through a feedback mechanism, hyperprolactinaemia can directly enhance dopamine levels (52). Therefore, an increase in dopamine through the tuberoinfundibular pathway mediated by hyperprolactinaemia could also trigger psychotic symptoms (25), enhancing the hypothesis that prolactin could be one among many causes and not a side effect of psychosis. Of note, patients with CT were older at the beginning of the follow-up, which could be associated with CT being associated with delays in help-seeking, explaining a previously reported older age at entry into the program (55).

At baseline, CT was unexpectedly associated with a diminished risk of presenting non-HDL levels above 2.8 mmol/L. Non-HDL levels were not examined in previous studies in FEP with CT (8, 20). Compared with healthy subjects, multiple FEP studies showed lower TC and LDL levels, assuming a better overall metabolic profile (6–9), which is also unexpected considering the high rates of metabolic syndrome in FEP (3). Interestingly, a study on first-episode schizophrenia with CT found higher TC and LDL levels and decreased HDL levels compared to first-episode schizophrenia without CT. Nevertheless, these differences did not remain significant after corrections for multiple testing (N=83) (22). CT in the general adult population has a negative impact on metabolic parameters (12); hence, an apparent protection of CT on non-HDL levels at baseline could be a chance finding. Of note, in the present study, CT was associated with stronger increases in non-HDL levels at 12 months, suggesting that CT increases metabolic alteration induced by antipsychotics. This is in agreement with CT being previously associated with increased waist circumference in the same FEP cohort treated with antipsychotics (21).

Interestingly, CT and prolactin enhance the response of the HPA axis to stress by increasing glucocorticoid receptors and adrenal cortex sensitivity, respectively, thereby indirectly acting on metabolism. In addition, hyperprolactinaemia has also been found to act directly on lipid synthesis, leading to hypercholesterolaemia and hypertriglyceridaemia (56, 57). Moreover, prolactin has been found to promote inflammation (58), which is a risk factor for metabolic alterations, particularly glucose intolerance (56). Thus, CT and prolactin could be part of the explanation of metabolic alterations in a FEP population.

In the general population, adverse parental-rearing practices and poor maternal responsiveness in early infancy were associated with lower levels of HDL cholesterol in young adulthood, but no association was demonstrated for other cardiometabolic parameters (LDL, TC, triglycerides) (36). In another study, an increased metabolic risk was found in middle-aged men with a conflicted mother-son relationship in childhood. This risk was diminished in women with a positive father-daughter relationship, which corroborates our results (35). On a biological level, maternal relationship quality has been recently linked to epigenetic modifications and telomere length, affecting stress regulation and metabolic risk (59). We should, however, first stress that a single question about the quality of the relationship with parents asked at baseline can neither be considered a reliable evaluation of the parent-child interaction during childhood nor an objective reflection of the education received before illness onset. We nevertheless observed that patients reporting LGRF were associated with a worse metabolic outcome during treatment. Thus, LGRF was significantly associated with stronger increases of LDL and non-HDL at 2 and 12 months, and of TC at 12 months.

No association was found between CT, LGRM, nor LGRF with FPG at baseline. Contradictory results have been published, with an increase in HbA1c, insulin, and C-peptide measured in CT patients, making them at risk for FPG disorder and eventually diabetes (22, 60), while another study did not find any effect of CT on HbA1c when comparing FEP patients with CT to healthy controls (20). Because the present study was not able to analyse FPG throughout the first year due to drop-outs during long-term follow-up, this issue should be addressed in future studies.

Overall, the present study supports the concept that CT, LGRF, or LGRM could act as risk factors for metabolic dysregulation under psychotropic medication, thus explaining how most of our positive associations appeared after several months. In addition, LGRM was associated with higher levels of prolactinaemia at baseline in women.

### 4.1 Strengths and limitations

This study has several limitations and strengths. First, this is an observational, longitudinal study, which does not allow for establishing a causal relationship. Second, patients with GRF or GRM were more likely to drop out of the study, an unexpected finding as secure attachment has been associated with greater compliance with treatment and follow-up (61). This could have introduced a selection bias with metabolic alterations observed in patients with LGRF or LGRM because of the long follow-up period. Third, because of the moderate sample size, not all relevant covariates were included in the analysis (i.e., Positive and Negative Syndrome Scale (PANSS) scores for prolactin or waist circumference for metabolic parameters). Fourth, prolactin analyses were made in non-fasting patients regardless of their menstrual cycle. Analyses of prolactin levels were also performed without excluding macroprolactin, which could have increased the rate of elevated levels of prolactin. Nonetheless, a recent study showed that hyperprolactinaemia in women with psychotic disorders was not significantly related to the presence of macroprolactin (62). Fifth, the definition of quality of relationship with parents was not assessed on a standardized scale, thus making it difficult to compare with previous studies. Given this, the results should therefore be interpreted with caution. Finally, the results presented here considered only relationships with parents and did not take other potential relationships into account.

Strengths of the present study include the longitudinal follow-up after antipsychotic initiation, enabling the prospective assessment of CT, LGRF, and LGRM effects on prolactin and metabolic parameters in real-life conditions.

## 5 Conclusion

The present study showed that a history of CT, which includes forms of sexual, physical, and emotional abuse or lack of a good relationship with one parent, was associated with worse metabolic and endocrine profiles in FEP patients following treatment with antipsychotics. Relationships with parents in FEP patients should be studied more in depth using standardized scales. Considering the health consequences of such metabolic and endocrine modifications, future studies with larger cohorts should be performed to better define such associations and their mediating mechanisms. Furthermore, investigating the biological pathways involved, particularly the potential role of epigenetic processes linking environmental and psychosocial risk factors to metabolic alterations, represents a promising direction for future research.

## Data Availability

Data from PsyMetab cannot be publicly deposited due to participant confidentiality purposes. Data from PsyMetab can be accessed after formal application and ethical review by the Ethics Committee of the Canton of Vaud. For further details: https://www.chuv.ch/cnp-psymetab. Similarly, due to ethical restrictions, the TIPP data used in the current study are not publicly available. Interested parties may request access from Sandra.Vieira@chuv.ch through a reasonable inquiry, subject to approval by the Regional Ethics Committee.
