# Supplementary material for: Childhood trauma and relationship with the parents: associations with lipids, glucose, and prolactin levels during antipsychotic treatment in patients with first-episode psychosis
Source: Front Psychiatry. 2025 Aug 14;16:1627203. doi: 10.3389/fpsyt.2025.1627203 (PMC12391041; doi:10.3389/fpsyt.2025.1627203)
Supplement: Supplementary file 1 [file Supplementaryfile1.docx]

## Supplementary information on clinical assessment of CT and quality of relationship with mother / father

Childhood trauma :

| **1.Traumatic event ?** _____ 0 No 1 Yes | | | | | |
| --- | --- | --- | --- | --- | --- |
| **2. If yes, which one ? (0= no, 1 = yes)** | Premorbid | Prodrome | After beginning of psychotic symptoms | Repeated (no / yes) | Age |
| Sexual abuse | 0 1 | 0 1 | 0 1 | 0 1 |  |
| Physical abuse | 0 1 | 0 1 | 0 1 | 0 1 |  |
| Emotional abuse | 0 1 | 0 1 | 0 1 | 0 1 |  |
| Note : “Repeated” assumes only premorbid period | | | | | |
| Premorbid : before any psychiatric disease | | | | | |
| Prodrome : presence of psychiatric symptoms but not psychotic symptoms | | | | | |

Clinicians at TIPP are trained to conduct an extensive assessment of patients, including evaluation of exposure to traumatic life events. Case managers meet patients frequently over the treatment period, which provides the framework to establish a trusting relationship, where extensive knowledge of patients’ history can be gathered. If patients agree, information can also be completed with family. In the case of inconsistency between the patient’s and the family’s report or in case of doubt regarding the exposure to trauma or the age at the time of exposure, patients were not included in the study (Alameda et al., 2015) Case managers complete a table during the patients’ 3 years of treatment, where exposure to traumatic life events can be recorded as follows: [1] Type of traumatic life event, rated as present or absent [sexual abuse, physical abuse, emotional and physical neglect, emotional abuse, among others…]; [2] time of occurrence in relation to psychosis stage [during the premorbid phase, during the prodrome or after onset of psychosis]; [3] age at the time of first exposure to each one of the traumas that occurred; and [4] single or repeated exposure to each one of the traumas that occurred. Considering that the clinicians who assessed exposure to life events did not rate the subjective perception of severity of the different forms of stressful events, patients were considered traumatized if they had been exposed to at least one experiences of abuse [physical, sexual, or emotional]. The consideration was that such events would undoubtedly be considered as highly traumatizing by anyone, and have been shown to be associated with risk for psychosis and functional deficits in psychotic samples (Varese et al., 2012) Sexual abuse refers to sexual molestation and/or rape. Physical abuse refers to physical attack or assault, or being repetitively beaten by parents, relatives, or caregivers. Emotional abuse was defined as verbal assaults on a child’s sense of worth or well-being or any humiliating or demeaning behavior directed toward a child by an adult or older person.

Relationship with parents :

On the first meeting, assessment of relationship with every member of the nuclear family has been range from 0 to 5 :

0= none ; 1 = very bad ; 2 = bad ; 3 = acceptable ; 4 = good ; 5 = excellent

Father : ___

Mother : ___

Brother/sister : ___

Brother/sister : ___

Brother/sister : ___

For the purpose of this study, we dichotomized this variable into presence of good relationship (ranges 3 to 5) or lack of good relationship of good relationship with father/mother (0 to 2).

## Supplementary information on ethnicity

Ethnicity was based on clinical data from both follow-ups (PsyMetab and PsyClin) (African / African-American, Arabic, Asian, Caucasian, others) and missing values were filled in with data from the Genome-Wide Association Studies (GWAS) analyses performed in participants in PsyMetab (Perry et al., 2022). Because of the modest number of included patients, ethnicity was dichotomized into “Westerners” (Caucasian and Arabic from clinical data; European from GWAS) and “Not Westerners” (African/African-American, Asian, others from clinical data; African, East-Asian, Native American from GWAS).

## Supplementary Table 1

Psychotropic medication categorized by risk of weight gain

| **High risk** | **Medium risk** | **Low risk** |
| --- | --- | --- |
| Clozapine | Amitriptyline | Amisulpride |
| Olanzapine | Levomepromazine | Aripiprazole |
| Valproate | Lithium | Flupentixol |
|  | Mirtazapine | Haloperidol |
|  | Quetiapine | Lurasidone |
|  | Risperidone/Paliperidone | Promazine |
|  | Zuclopenthixol |  |

*No subject was receiving brexpiprazole nor cariprazine.*

## Supplementary Table 2A

Doses per antipsychotic chosen for weighting, adapted from Swissmedic recommendation

| **First-generation antipsychotic** | **Upper limit of the average** **dose per day** |
| --- | --- |
| Clotiapine | 200 mg |
| Flupentixol | 40 mg |
| Haloperidol | 20 mg |
| Levomepromazine | 250 mg |
| Zuclopenthixol | 75 mg |

| **Second-generation antipsychotic** | **Upper limit of the average** **dose per day** |
| --- | --- |
| Amisulpride | 800 mg |
| Aripiprazole | 30 mg |
| Clozapine | 600 mg |
| Lurasidone | 160 mg |
| Olanzapine | 20 mg |
| Paliperidone | 12 mg |
| Quetiapine | 800 mg |
| Risperidone | 10 mg |
| Risperidone (depot) | 50 mg / 2 weeks |

| **Non-antipsychotic** | **Upper limit of the average** **dose per day** |
| --- | --- |
| Lithium | 24 mmol/L |
| Valproate | 2000 mg |

*No subject was receiving brexpiprazole nor cariprazine.*

Weighting of the total daily dose of antipsychotic: Example of a fictional patient with 2 antipsychotics with weighting of total daily dose and the sum of weightings.

|  | Dose | Upper limit of the average dose per day of antipsychotic | Weighting of the total daily dose of antipsychotic (dose/upper limit of the average dose per day) | Sum of weightings of each antipsychotic taken in the same day |
| --- | --- | --- | --- | --- |
| Risperidone | 5 | 10 | 0.5 | 0.67 |
| Aripiprazole | 5 | 30 | 0.17 |  |

## Supplementary Table 3

Effect of antipsychotic on prolactinaemia according to Huhn et al. (Huhn et al., 2019)

| **First-generation antipsychotic** | **MD [ng/ml]*** |
| --- | --- |
| Clotiapine | *No data* |
| Flupentixol | -10.45 |
| Haloperidol | 18.49 |
| Levomepromazine | 8.7 (based on chlorpromazine MD) |
| Zuclopenthixol | *No data* |

| **Second-generation antipsychotic** | **MD [ng/ml]*** |
| --- | --- |
| Amisulpride | 26.87 |
| Aripiprazole | -7.10 |
| Clozapine | -77.05 |
| Lurasidone | 7.04 |
| Olanzapine | 4.47 |
| Paliperidone | 48.51 |
| Quetiapine | -1.17 |
| Risperidone | 37.98 |

*Adapted from Huhn M, Nikolakopoulou A, Schneider-Thoma J, Krause M, Samara M, Peter N, et al. Comparative efficacy and tolerability of 32 oral antipsychotics for the acute treatment of adults with multi-episode schizophrenia: a systematic review and network meta-analysis. The Lancet. 2019 Sep 14;394(10202):939–51.*

*Mean difference (MD) of prolactin elevation compared to placebo

Each antipsychotic was defined using the mean difference (MD) listed which represents in ng/ml the mean difference of prolactin elevation compared to placebo. If MD was less than 0, a value of 0 was attributed.

Antipsychotic not registered (no data) in the above-mentioned study and those with MD <0 were referenced “0” in prolactin effect.

## Supplementary List 1

Variables were analysed once on a continuous and once on a categorical scale, dichotomized by the median of the cohort. Finally, the percentage of differences in metabolic and hormonal parameters between groups (CT and non-CT, quality of relationship with the parent) were analysed between baseline and 2, 3, and 12 months of follow-up.

Models with metabolic variables were adjusted for potential covariates and confounders based on common cardiometabolic risk factors (age, sex, socio-economic status (Panczak et al., 2012), ethnicity, baseline BMI, smoking status, alcohol status, weight gain risk of psychotropic medication and weighting of total daily dose) (Delacrétaz et al., 2018). Models with prolactinaemia were adjusted for potential covariates and confounders (age, sex, medication during the follow up and weighting of the total daily dose).

Covariates used in linear models presented in Figure 1 (A-C):

1. LDL (low-density lipoprotein): models at baseline were adjusted for age and BMI baseline; models at 2 months were adjusted for age, BMI baseline, ethnicity, antipsychotic medication and smoking status; models at 12 months were adjusted for age, BMI baseline and antipsychotic medication. Medians of the cohort: 2.4 mmol/L at baseline, 2.58 mmol/L at 2 months, 2.83 mmol/L at 12 months.

Non-HDL: models at baseline were adjusted for age and BMI baseline; models at 2 months were adjusted for age and sex; models at 12 months were adjusted for age, sex, BMI baseline and antipsychotic medication. Medians of the cohort: 2.8 mmol/L at baseline, 3.1 mmol/L at 2 months, 3.4 mmol/L at 12 months.

1. HDL (high-density lipoprotein): models at baseline were adjusted for age, sex, ethnicity, and antipsychotic medication; models at 2 months were adjusted for sex and weighting of the total daily dose of antipsychotic; models at 12 months were adjusted for sex, BMI at baseline and weighting of the total daily dose of antipsychotic.

Non-HDL (non-high-density lipoprotein): models at baseline were adjusted for age, sex, BMI (body mass index) baseline, antipsychotic medication; models at 2 months were adjusted for age, sex and BMI baseline; models at 12 months were adjusted for age and sex.

TC (total cholesterol): models at baseline were adjusted for age and BMI baseline; models at 2 months were adjusted for age and sex; models at 12 months were adjusted for age and antipsychotic medication.

1. LDL: models at 2 months were adjusted for BMI baseline, smoking status, and antipsychotic medication; models at 12 months were adjusted for age and sex.

Non-HDL: models at 2 months were adjusted for BMI baseline, smoking status, and weighting of the total daily dose of antipsychotic for CT (childhood trauma) and LGRM (lack of good relationship with the mother). The model at 2 months for LGRF (lack of good relationship with the father) was adjusted for age, sex, BMI baseline and weighting of the total daily dose of antipsychotic; models at 12 months were adjusted for age, alcohol status and weighting of the total daily dose of antipsychotic.

TC: models at 2 months were adjusted for weighting of the total daily dose of antipsychotic and smoking status; models at 12 months were adjusted for sex, alcohol status and weighting of the total daily dose of antipsychotic for LGRF and LGRM. Model at 12 months for CT was adjusted for age, sex, weighting of the total daily dose of antipsychotic.

Covariates used in linear models presented in Figure 2 (A-B):

1. Models at baseline were adjusted for age, effect of antipsychotic medication on levels of prolactin and weighting of the total daily dose of antipsychotic for CT and LGRM. Model at baseline for LGRF was adjusted for effect of antipsychotic medication on levels of prolactin and weighting of the total daily dose of antipsychotic.
2. Models at baseline were adjusted for age and effect of antipsychotic medication on levels of prolactin for CT and LGRM. Model for LGRF was adjusted for effect of antipsychotic medication on levels of prolactin and weighting of the total daily dose of antipsychotic.

## Reference

Alameda, L., Ferrari, C., Baumann, P. S., Gholam-Rezaee, M., Do, K. Q., & Conus, P. (2015). Childhood sexual and physical abuse: Age at exposure modulates impact on functional outcome in early psychosis patients. *Psychological Medicine*, *45*(13), 2727–2736. https://doi.org/10.1017/S0033291715000690

Delacrétaz, A., Vandenberghe, F., Gholam-Rezaee, M., Saigi Morgui, N., Glatard, A., Thonney, J., Solida-Tozzi, A., Kolly, S., Gallo, S. F., Baumann, P., Berney, S., Zulauff, S. V., Aubry, J.-M., Hasler, R., Ebbing, K., von Gunten, A., Conus, P., & Eap, C. B. (2018). Early changes of blood lipid levels during psychotropic drug treatment as predictors of long-term lipid changes and of new onset dyslipidemia. *Journal of Clinical Lipidology*, *12*(1), 219–229. https://doi.org/10.1016/j.jacl.2017.10.002

Huhn, M., Nikolakopoulou, A., Schneider-Thoma, J., Krause, M., Samara, M., Peter, N., Arndt, T., Bäckers, L., Rothe, P., Cipriani, A., Davis, J., Salanti, G., & Leucht, S. (2019). Comparative efficacy and tolerability of 32 oral antipsychotics for the acute treatment of adults with multi-episode schizophrenia: A systematic review and network meta-analysis. *The Lancet*, *394*(10202), 939–951. https://doi.org/10.1016/S0140-6736(19)31135-3

Panczak, R., Galobardes, B., Voorpostel, M., Spoerri, A., Zwahlen, M., Egger, M., & Swiss National Cohort and Swiss Household Panel. (2012). A Swiss neighbourhood index of socioeconomic position: Development and association with mortality. *Journal of Epidemiology and Community Health*, *66*(12), 1129–1136. https://doi.org/10.1136/jech-2011-200699

Perry, B. I., Vandenberghe, F., Garrido-Torres, N., Osimo, E. F., Piras, M., Vazquez-Bourgon, J., Upthegrove, R., Grosu, C., De La Foz, V. O.-G., Jones, P. B., Laaboub, N., Ruiz-Veguilla, M., Stochl, J., Dubath, C., Canal-Rivero, M., Mallikarjun, P., Reymond-Delacrétaz, A., Ansermot, N., Fernandez-Egea, E., … Crespo-Facorro, B. (2022). The psychosis metabolic risk calculator (PsyMetRiC) for young people with psychosis: International external validation and site-specific recalibration in two independent European samples. *The Lancet Regional Health - Europe*, *22*, 100493. https://doi.org/10.1016/j.lanepe.2022.100493

Varese, F., Smeets, F., Drukker, M., Lieverse, R., Lataster, T., Viechtbauer, W., Read, J., van Os, J., & Bentall, R. P. (2012). Childhood adversities increase the risk of psychosis: A meta-analysis of patient-control, prospective- and cross-sectional cohort studies. *Schizophrenia Bulletin*, *38*(4), 661–671. https://doi.org/10.1093/schbul/sbs050
